# Correlation Among Psychological Resilience, Social Support, and Coping Style in Patients With Complicated Hepatolithiasis

**DOI:** 10.3389/fnbeh.2022.939512

**Published:** 2022-06-15

**Authors:** Ying Wang, Chunqiu Huang, Honghui Zhang, Yimin Cai, Zhoumin Shen, Xiahong Hu, Bifang Zhou, Lifen Yang, Qian Chen

**Affiliations:** Hunan Provincial People’s Hospital (The First-Affiliated Hospital of Hunan Normal University), Changsha, China

**Keywords:** medical behavior, complicated hepatolithiasis, psychological resilience, social support, coping style

## Abstract

**Objectives**: To investigate the correlation between psychological resilience, social support, and coping styles in patients with complicated hepatolithiasis.

**Methods**: The objective sampling method was used in this study to select a total of 156 patients with complicated hepatolithiasis in a Third-class Grade A hospital in Changsha, Hunan Province from January to December 2019. Self-designed general data questionnaire, the Connor-Davidson Resilience Scale (CD-RISC), the Social Support Rating Scale (SSRS), and the Simplified Coping Style Questionnaire (SCSQ) were used to conduct a questionnaire investigation. Spearman correlation analysis was used to analyze the correlation between psychological resilience, social support, and coping styles in patients with complicated hepatolithiasis.

**Results**: Both the total score of psychological resilience (45.79 ± 16.28) and social support (35.71 ± 9.92) of patients with complicated hepatolithiasis were significantly lower than those of the domestic norm. The total score of psychological resilience of patients with complicated hepatolithiasis was positively correlated with the total score of social support (*r* = 0.570, *p* < 0.01). The total score of psychological resilience and its three dimensions were positively correlated with the positive coping (*r* = 0.682, 0.673, 0.663, 0.535, *p* < 0.01)and negatively correlated with negative coping (*r* = −0.240, −0.207, −0.221, −0.286, *p* < 0.01).

**Conclusions**: This study indicated that strengthening social support and improving the coping style of patients with complicated hepatolithiasis are helpful to improve their psychological resilience, which provided theory basis and reference for further intervention measures to improve social support system and coping styles.

## Introduction

With the continuous improvement of people’s living standard and change of dietary structure and lifestyle of residents, hepatolithiasis has become an important reason for the death of benign biliary tract diseases in China (Iwashita et al., 2016; Chen et al., 2018). Hepatolithiasis, that is, primary hepatolithiasis, refers to the stone formation in the intrahepatic biliary system (Lorio et al., 2020; Xiao et al., 2021). Global epidemiology shows that the incidence rate of hepatolithiasis is as high as 30%–50% in Asian countries such as China, South Korea and Japan, but relatively low in other Western countries (Nakayama et al., 1986; Pausawasdi and Watanapa, 1997; Kayhan et al., 2007). At present, the central link of the treatment principles of hepatolithiasis is removing primary lesions and the main treatment method is surgical treatment (Ambreen et al., 2009). Patients with complicated hepatolithiasis suffer from progressive injury of the hepatobiliary duct due to repeated attacks of cholangitis. In addition, patients are complicated with terminal biliary diseases such as portal hypertension, biliary cirrhosis, and cholangiocarcinoma, which is a major problem in the research field of hepatobiliary surgery and liver transplantation (Suzuki et al., 2014). It has been reported a high residual rate and recurrence rate of hepatolithiasis patients after an operation with a long course of the disease and many complications, resulting in multiple surgical treatments of patients (Cheon et al., 2009; Zhang et al., 2015).

Both physiological and psychological problems of patients caused by repeated surgery and surgical pain resulted in a great negative impact on the quality of life of patients (Badner et al., 1990; Kayabasi et al., 2021). Preoperative anxiety is one of the important causes of stress reaction, which can affect patients’ body health. A bad psychological status may have a negative effect on lowering immune function, promoting catabolism, inhibiting gastrointestinal peristalsis, and increasing cardiopulmonary burden, so as to greatly affects the normal recovery of patients (Jamison et al., 1987). In recent years, positive psychology has gradually developed. As a new breakthrough point, resilience has become a research hotspot in the field of mental health at home and abroad and has been widely used in various fields such as psychology, pedagogy, military affairs, cancer, and patients with chronic diseases (Barthélemy et al., 2021; Talubo et al., 2022). Resilience is one of the important reasons for maintaining mental health, which refers to the ability of individuals to confront and adapt to stress, adversity, or trauma (Southwick and Charney, 2012). It plays an important role in resisting major stress events, promoting individual psychological recovery, and improving their quality of life (Morote et al., 2017; McGowan et al., 2018).

Social support is the emotional and material help and support that people get from all walks of life, including families, relatives, friends, and other organizations, which is the external protection factor of mental resilience and can significantly promote psychological health (Cohen, 2004; Gariépy et al., 2016; Gong and Kim, 2017). Coping style refers to an individual’s cognitive and behavioral efforts to meet internal and external needs, which is classified into positive coping and negative coping (Ellis et al., 2016). Studies by Yu et al. (2013) show that patients’ anxiety, depression, and other bad psychological states can be improved by positive coping styles. Positive coping can reduce the negative impact of various stress events on patients’ psychology and cognition so as to improve their quality of life.

At present, the previous research mainly focuses on the perioperative nursing, treatment plan, and negative emotions of patients with complicated hepatolithiasis. However, this study investigates their psychological resilience from the perspective of positive psychology. In addition, few scholars have studied psychological resilience, social support, and coping style of patients with complicated hepatolithiasis, and there is no relevant report on the association among them. In this study, the correlations among psychological, social support, and coping styles of patients with complicated hepatolithiasis were researched and analyzed, which also provide a reference for the recovery and improvement of psychological resilience of patients with complicated hepatolithiasis.

## Objective and Methods

### Research Objects

A total of 156 patients with complicated hepatolithiasis in a Third-class Grade A hospital in Changsha, Hunan Province from January to December 2019 were selected as the research objects.

#### Inclusion Criteria

(1) Age ≥ 18 years old.
(2) Patients with complicated hepatolithiasis who meet the diagnostic criteria.
(3) Those who can complete the questionnaire independently or with the help of researchers.
(4) Those who can communicate normally and voluntarily participate in this survey and sign the informed consent form.

#### Exclusion Criteria

(1) Patients with malignant tumors other than the biliary system or acute and critical diseases such as severe cardiac, pulmonary, and renal insufficiency or patients with terminal diseases.
(2) Those who encounter major life events within half a year.
(3) Patients or their family members are unwilling to cooperate with the research and quit halfway.

### Research Methods

This study is a descriptive study. A questionnaire survey was conducted on patients with complicated hepatolithiasis who met the inclusion criteria by means of objective sampling. The main research tools were used in this study: a general information questionnaire, the Connor-Davidson Resilience Scale (CD-RISC), the Social Support Rating Scale (SSRS), and the Simplified Coping Style Questionnaire (SCSQ).

### Survey Tools

#### General Information Questionnaire

The general information questionnaire included the patient’s gender, age, religious belief, as well as the number of operations, postoperative catheter management, and other disease-related clinical data.

#### Connor-Davidson Resilience Scale (CD-RISC)

American expert Connor and other experts (Connor and Davidson, 2003) designed it in 2003, which was translated and revised by Yu and Zhang (2007) according to the cultural background of China. The scale has good reliability and validity with Cronbach’s *α* = 0.91. It is divided into three dimensions and 25 items, namely, tenacity, strength, and optimism. The Likert 5-grade evaluation method was used, that is, “almost always like this”, “often like this”, “sometimes like this”, “rarely like this” and “not like this at all”. 4 points, 3 points, 2 points, 1 point, and 0 points were given respectively, with a total score of 0 – 100 points. The scores of each item were added up. The higher the cumulative score, the better the psychological resilience.

#### Social Support Rating Scale (SSRS)

This scale was designed based on Xiao’s (1994) research. Cronbach’s α coefficient is 0.94 with high test-retest reliability. It contains three dimensions, namely, objective support, subjective support, and support utilization. According to the score, it can be divided into three levels: 45–66 is a high level, 23–44 is a medium level, and ≤22 is a low level. The score indicates the high and low level of social support respectively.

#### Simplified Coping Style Questionnaire (SCSQ)

This scale is compiled by Xie (1998) combining Chinese cultural background and summarizing the research experience at home and abroad. The Cronbach’s α of this scale is 0.90 with good reliability and validity. There are 20 questions, including positive and negative dimensions. Comparing the items of the two aspects, high average score of the items indicates that the research subjects mainly adopt corresponding coping styles. This scale has been widely used in all kinds of people.

### Statistical Methods

SPSS 21.0 software was used for data collation and statistical analysis. *T*-test and variance analysis were used to analyze variables that conform to normal distribution. Spearman correlation analysis was used to analyze statistical data on the correlation between psychological resilience, social support, and coping style. *P* < 0.05 indicates that the difference is statistically significant.

## Results and Discussion

In this study, a total of 158 questionnaires were sent out, of which 156 were valid. The effective recovery rate was 98.7%. There are 73 males (46.8%) and 83 females (53.2%), with the average age of 52.98 ± 12.82 years old. General information of patients with complicated hepatolithiasis were shown in Table 1.

Table 2 showed that the total score of psychological resilience of patients with complicated hepatolithiasis is 45.79 ± 16.28. The scores of each dimension from high to low are tenacity (23.30 ± 9.19), strength (15.37 ± 5.35), and optimism (7.12 ± 2.69) respectively. Compared with the domestic norm, both the total score of psychological resilience and all dimensions were significantly lower, as shown in Table 3. The results are consistent with the previous report (Yu and Zhang, 2007), which suggested that clinical medical staff and patients’ families should pay attention to the intervention and improvement of resilience of patients with complicated hepatolithiasis.

As shown in Table 4, the total score of social support in patients with complicated hepatolithiasis is 35.71 ± 9.92, which is in the middle level. The scores of each dimension from high to low are subjective support (21.22 ± 5.53), objective support (8.26 ± 3.71), and degree for support utilization (6.22 ± 2.11). As shown in Table 5; the total score of social support and scores of all dimensions are lower than the domestic norm.

**Table 1:** General information of research objects (*n* = 156, %).

Variables	Category	Frequency	Percentage (%)
Gender	Males	73	46.8
	Females	83	53.2
Age	<45 years old	39	25.0
	45<60 years old	75	48.1
	>60 years old	42	26.9
Religious belief	Yes	17	10.9
	No	139	89.1
Family residence	Village	95	60.9
	Cities and town	61	39.1
Marital status	Unmarried	13	8.3
	Married	123	78.9
	Divorced or widowed	20	12.8
Monthly household income (RMB)	<1,000	35	22.4
	1,000<3,000	50	32.1
	3,001<5,000	41	26.3
	>5,000	30	19.2
Educational level	Primary school or below	39	25.0
	Junior high school	40	25.6
	High school/technical secondary school	41	26.3
	universities and colleges	19	12.2
	Bachelor degree or above	17	10.9
Payment ways	Self-funded	33	21.2
	The new rural cooperative medical insurance	49	31.4
	Medical insurance for urban residents	62	39.7
	Other ways	12	7.7
Occupations	Worker	19	12.2
	Farmer	54	34.6
	Professional and technical personnel	18	11.5
	Cadre	6	3.9
	retiree	22	14.1
	Unemployed or other	37	23.7
Disease course (years)	<3	51	32.7
	3<5	54	34.6
	6<10	23	14.8
	>10	28	17.9
Operation time(s)	≤1	29	18.6
	2<3	104	66.7
	≥4	23	14.7
Postoperative catheter	Yes	145	92.9
	No	11	7.1
Complicated with disease	Yes	81	51.9
	No	75	48.1
Knowledge of the disease	Know about all	22	14.1
	Know partially	122	78.2
	Know nothing	12	7.7

**Table 2:** Total scores and score of each dimension of psychological resilience in patients with complicated hepatolithiasis (*n* = 156).

Dimensions	Items	Minimum	Maximum	Average
		value	value	
Tenacity	13	4.00	52.00	23.30 ± 9.19
Strength	8	2.00	32.00	15.37 ± 5.35
Optimism	4	1.00	16.00	7.12 ± 2.69
Total scores	25	7.00	96.00	45.79 ± 16.28

**Table 3:** Comparison of psychological resilience level in patients with complicated hepatolithiasis and domestic norm (*n* = 156, x¯ ± s).

Dimensions	Scores of	Domestic	*t*	*p*
	patients (*n* = 156)	norm (*n* = 560)		
Tenacity	23.30 ± 9.19	31.26 ± 4.21	−10.82	<0.001**
Strength	15.37 ± 5.35	23.31 ± 4.64	−18.54	<0.001**
Optimism	7.12 ± 2.69	9.56 ± 3.33	−11.33	<0.001**
Total scores	45.79 ± 16.28	65.40 ± 13.90	−15.04	<0.001**

**p < 0.01.

**Table 4:** Total score of social support and score of each dimension in patients with complicated hepatolithiasis (*n* = 156).

Dimensions	Items	Minimum	Maximum	Average
		value	value	
Objective support	3	1.00	18.00	8.26 ± 3.71
Subjective support	4	8.00	32.00	21.22 ± 5.53
Degree for	3	3.00	12.00	6.22 ± 2.11
support utilization
Total score	10	12.00	60.00	35.71 ± 9.92

**Table 5:** Comparison of social support in patients with complicated hepatolithiasis and domestic norms (*n* = 156, x¯ ± s).

Dimensions	Scores of patients (*n* = 156)	Domestic norm (*n* = 1,158)	*t*	*p*
Objective support	8.26 ± 3.71	12.68 ± 3.47	−14.86	<0.001**
Subjective support	21.22 ± 5.53	23.81 ± 4.75	−5.85	<0.001**
Degree for support utilization	6.22 ± 2.11	9.38 ± 2.40	−18.67	<0.001**
Total score	35.71 ± 9.92	44.34 ± 8.38	−10.88	<0.001**

**p < 0.01.

Among the three dimensions of social support, the score of subjective support is the highest. Patients’ support is mainly provided by family members such as spouses, parents, and children, indicating that support from family members plays an important role in social support. The objective support score is low, indicating that patients receive very little practical help from the government, charitable organizations, and various welfare organizations. A low degree of support utilization indicates that patients cannot make full use of the surrounding resources when facing the problems caused by diseases and medical treatments. Therefore, we should pay more attention to the proper utilization of patients’ social support system and take active measures to provide practical help to patients in emotional and material aspects.

As shown in Table 6, scores of coping styles of patients with complicated hepatolithiasis are positive coping style (1.40 ± 0.63) and negative coping style (1.52 ± 0.57), respectively. The score of negative coping style is higher than that of the positive coping style, which indicates that patients with complicated hepatolithiasis tend to adopt negative coping style. Scholars’ research shows that long-term recurrence of chronic diseases can easily lead to patients’ negative coping styles and affect their self-care ability (Rechenberg et al., 2017), which is consistent with the result of Table 6. A study shows that the stronger a patient’s coping ability, the higher their quality of life, which has been proved to be able to predict patient’s self-care ability (Anderson et al., 2017). Therefore, medical staff should be more focused on the propagation of relevant medical knowledge and encourage patients to actively cooperate with all kinds of diagnosis and treatment nursing by dynamically evaluating their coping styles.

**Table 6:** Total score of coping styles and score of each dimension in patients with complicated hepatolithiasis (*n* = 156).

Dimensions	Items	Minimum	Maximum	Average
		value	value	
Positive coping	12	1.00	36.00	1.40 ± 0.63
Negative coping	8	2.00	24.00	1.52 ± 0.57

As shown in Table 7, Spearman correlation analysis shows that both total scores and all dimensions of psychological resilience of 156 patients with complicated hepatolithiasis are positively correlated with those of social support (*p* < 0.01), which indicated that the more social support patients receive, the higher their psychological resilience level. This is similar to the previous research results (Donnellan et al., 2015). Therefore, it is necessary to evaluate the social support system of patients with complicated hepatolithiasis. According to their social support level and situation, family members and friends should be guided to give more spiritual and material support to patients. Encouraging patients to actively participate in social activities, broadening their circle of friends, and making better use of the social support system is helpful to improve their psychological resilience.

**Table 7:** Correlation between total scores of psychological resilience and social support in patients with complicated hepatolithiasis (*r*).

Dimensions	Tenacity	Strength	Optimism	Total score of social support
Objective support	0.389**	0.442**	0.368**	0.428**
Subjective support	0.541**	0.543**	0.457**	0.562**
Degree for support utilization	0.347**	0.328**	0.302**	0.352**
Total score of psychological resilience	0.545**	0.557**	0.467**	0.570**

Table shows the correlation coefficient, **p < 0.01.

From Table 8, it can be seen that total scores and each dimension of psychological resilience are positively correlated with positive coping (*p* < 0.01) and negatively correlated with negative coping (*p* < 0.01), which is consistent with Wang et al.’s (2016) research. Results show that patients with better psychological resilience are more likely to adopt positive coping styles when facing difficulties. Some studies have also confirmed that positive coping styles can relieve mental stress and promote psychological resilience, while negative coping styles will aggravate stress and negatively affect psychological resilience (Manne et al., 2015). For patients with complicated hepatolithiasis, medical staff should give correct guidance and introduce more about the positive effects of optimistic and positive emotions on the disease to promote their recovery.

**Table 8:** Correlation between total score of psychological resilience and coping styles in patients with complicated hepatolithiasis (*r*).

Dimensions	Tenacity	Strength	Optimism	Total scores of social support
Positive coping	0.673**	0.663**	0.535**	0.682**
Negative coping	−0.207**	−0.221**	−0.286**	−0.240**

Table shows the correlation coefficient, **p < 0.01.

## Conclusion

This study investigated the correlation between psychological resilience, social support, and coping styles of patients with complicated hepatolithiasis and expanded the research direction of the mental field of patients with complicated hepatolithiasis. Results indicated that strengthening social support and improving the coping style of patients with complicated hepatolithiasis are helpful to improve their psychological resilience. Additionally, this study arouses clinical medical staff’s attention to psychological resilience, social support and coping style of patients with complicated hepatolithiasis, which provides theoretical basis and references for further measures to improve psychological resilience level of patients with complicated hepatolithiasis and conduct corresponding intervention research.

## Data Availability Statement

The raw data supporting the conclusions of this article will be made available by the authors, without undue reservation.

## Ethics Statement

The studies involving human participants were reviewed and approved by Hunan Provincial People’s Hospital (The First-Affiliated Hospital of Hunan Normal University). The patients/participants provided their written informed consent to participate in this study.

## Author Contributions

YW and CH contributed to conception and design of the study, and wrote the first draft of the manuscript. HZ contributed to manuscript revision, reading, and project management. YC, ZS, XH, BZ, LY, and QC contributed to the data collection and analysis. All authors contributed to the article and approved the submitted version.

## Conflict of Interest

The authors declare that the research was conducted in the absence of any commercial or financial relationships that could be construed as a potential conflict of interest.

## Publisher’s Note

All claims expressed in this article are solely those of the authors and do not necessarily represent those of their affiliated organizations, or those of the publisher, the editors and the reviewers. Any product that may be evaluated in this article, or claim that may be made by its manufacturer, is not guaranteed or endorsed by the publisher.

## References

[B1] Ambreen M. Shaikh A. R. Jamal A. Qureshi J. N. Dalwani A. G. Memon M. M. (2009). Primary closure versus T-tube drainage after open choledochotomy. Asian J. Surg. 32, 21–25. 10.1016/S1015-9584(09)60004-X 19321398

[B2] Anderson B. J. Laffel L. M. Domenger C. Danne T. Phillip M. Mazza C.. (2017). Factors associated with diabetes-specific health-related quality of life in youth with type 1 diabetes: the global teens study. Diabetes Care 40, 1002–1009. 10.2337/dc16-1990 28546221 PMC5864137

[B3] Badner N. H. Nielson W. R. Munk S. Kwiatkowska C. Gelb A. W. (1990). Preoperative anxiety: detection and contributing factors. Can. J. Anaesth. 37, 444–447. 10.1007/BF03005624 2340614

[B4] Barthélemy E. J. Thango N. S. Höhne J. Lippa L. Kolias A. WFNS Young Neurosurgeons Forum Resilience Task Force. (2021). Resilience in the face of the COVID-19 pandemic: how to bend and not break. World Neurosurg. 146, 280–284. 10.1016/j.wneu.2020.11.105 33249221 PMC7836866

[B5] Chen W. Lou J. Liang T. (2018). Comparison of laparoscopic versus open left lateral hepatectomy for hepatolithiasis in China. HPB 20:S734. 10.1016/j.hpb.2018.06.1492

[B6] Cheon Y. K. Cho Y. D. Moon J. H. Lee J. S. Shim C. S. (2009). Evaluation of long-term results and recurrent factors after operative and nonoperative treatment for hepatolithiasis. Surgery 146, 843–853. 10.1016/j.surg.2009.04.009 19744434

[B7] Cohen S. (2004). Social relationships and health. Am. Psychol. 59, 676–684. 10.1037/0003-066X.59.8.676 15554821

[B8] Connor K. M. Davidson J. R. T. (2003). Development of a new resilience scale: the connor-davidson resilience scale (CD-RISC). Depress. Anxiety 18, 76–77. 10.1002/da.10113 12964174

[B9] Donnellan W. J. Bennet K. M. Soulsby L. K. (2015). What are the factors that facilitate or hinder resilience in older spousal dementia cancers? A qualitative study. Aging Ment. Health 19, 932–939. 10.1080/13607863.2014.977771 25410637

[B10] Ellis L. Gergen J. Wohlgemuth L. Nolan M. T. Aslakson R. (2016). Empowering the “cheerers”: role of surgical intensive care unit nurses in enhancing family resilience. Am. J. Crit. Care 25, 39–45. 10.4037/ajcc2016926 26724292

[B11] Gariépy G. Honkaniemi H. Quesnel-Vallée A. (2016). Social support and protection from depression: systematic review of current findings in Western countries. Br. J. Psychiatry 209, 284–293. 10.1192/bjp.bp.115.169094 27445355

[B12] Gong E. H. Kim W. Y. (2017). Meta-analysis of the factors that influence adolescent depression. J. Korean Soc. Wellness 12, 61–75. 10.21097/ksw.2017.08.12.3.61

[B13] Iwashita T. Nakai Y. Hara K. Isayama H. Itoi T. Park D. H. (2016). Endoscopic ultrasound-guided antegrade treatment of bile duct stone in patients with surgically altered anatomy: a multicenter retrospective cohort study. J. Hepatobiliary Pancreat. Sci. 23, 227–233. 10.1002/jhbp.329 26849099

[B14] Jamison R. N. Parris W. C. Maxson W. S. (1987). Psychological factors influencing recovery from outpatient surgery. Behav. Res. Ther. 25, 31–37. 10.1016/0005-7967(87)90112-4 2954531

[B16] Kayabasi S. Cayir S. Hizli O. (2021). The effects of intraday operation time on pain and anxiety of patients undergoing septoplasty. Braz. J. Otorhinolaryngol. 87, 310–314. 10.1016/j.bjorl.2019.09.006 31771818 PMC9422450

[B17] Kayhan B. Akdoğan M. Parlak E. Ozarslan E. Sahin B. (2007). Hepatolithiasis: a Turkey experience. Turk. J. Gastroenterol. 18, 28–32. Available online at: https://turkjgastroenterol.org/en/hepatolithiasis-a-turkey-experience-1621431. 17450492

[B18] Lorio E. Patel P. Rosenkranz L. Patel S. Sayana H. (2020). Management of hepatolithiasis: review of the literature. Curr. Gastroenterol. Rep. 22:30. 10.1007/s11894-020-00765-3 32383039

[B19] Manne S. L. Myers-Virtue S. Kashy D. Ozga M. Kissane D. Heckman C.. (2015). Resilience, positive coping and quality of life among women newly diagnosed with gynecological cancers. Cancer Nurs. 38, 375–382. 10.1097/NCC.0000000000000215 25521911 PMC4470889

[B20] McGowan J. A. Brown J. Lampe F. C. Lipman M. Smith C. Rodger A. (2018). Resilience and physical and mental well-being in adults with and without HIV. AIDS Behav. 22, 1688–1698. 10.1007/s10461-017-1980-6 29159595 PMC5902512

[B23] Morote R. Hjemdal O. Uribe P. M. Corveleyn J. (2017). Psychometric properties of the Resilience Scale for Adults (RSA) and its relationship with life-stress, anxiety and depression in a Hispanic Latin-American community sample. PLoS One 12:e0187954. 10.1371/journal.pone.0187954 29125876 PMC5681258

[B21] Nakayama F. Soloway R. D. Nakama T. Miyazaki K. Ichimiya H. Sheen P. C.. (1986). Hepatolithiasis in East Asia: retrospective study. Dig. Dis. Sci. 31, 21–26. 10.1007/BF01347905 3940820

[B22] Pausawasdi A. Watanapa P. (1997). Hepatolithiasis: epidemiology and classification. Hepatogastroenterology 44, 314–316. 9164496

[B24] Rechenberg K. Whittemore R. Holland M. Grey M. (2017). General and diabetes-specific stress in adolescents with type 1 diabetes. Diabetes Res. Clin. Pract. 130, 1–8. 10.1016/j.diabres.2017.05.003 28551480 PMC5608607

[B25] Southwick S. M. Charney D. S. (2012). The science of resilience: implications for the prevention and treatment of depression. Science 338, 79–82. 10.1126/science.1222942 23042887

[B26] Suzuki Y. Mori T. Yokoyama M. Nakazato T. Abe N. Nakanuma Y.. (2014). Hepatolithiasis: analysis of Japanese nationwide surveys over a period of 40 years. J. Hepatobiliary Pancreat. Sci. 21, 617–622. 10.1002/jhbp.116 24824191

[B15] Talubo J. P. Morse S. Devendra S. (2022). Whose resilience matters? A socio-ecological systems approach to defining and assessing disaster resilience for small islands. Env. Challenges 7:100511. 10.1016/j.envc.2022.100511

[B27] Wang L. J. Zhong W. X. Ji X. D. Chen J. (2016). Depression, caregiver burden and social support among caregivers of retinoblastoma patients in China. Int. J. Nurs. Pract. 22, 478–485. 10.1111/ijn.12458 27325472

[B28] Xiao’s S. Y. (1994). Theoretical base and research application of “social support rating scale”. J. Clin. Psychiatry 2, 98–100.

[B29] Xiao Z. Huang Z. Gao J. Wang J. Lei J. Zhou F.. (2021). The imbalance of biliary microflora in hepatolithiasis. Microb. Pathog. 157:104966. 10.1016/j.micpath.2021.104966 34023439

[B30] Xie Y. N. (1998). The preliminary study on the reliability and validity of the simplified coping style questionnaire. Chin. J. Clin. Psychol. 6, 114–115.

[B32] Yu Y. Hu J. Efird J. T. McCoy T. P. (2013). Social support, coping strategies and health-related quality of life among primary caregivers of stroke survivors in China. J. Clin. Nurs. 22, 2160–2171. 10.1111/jocn.12251 23829403

[B31] Yu X. N. Zhang J. X. (2007). Factor analysis and psychometric evaluation of the Connor-Davidson resilience scale (CD-RISC) with Chinese people. Soc. Behav. Personal. Int. J. 35, 19–30. 10.2224/sbp.2007.35.1.19

[B33] Zhang G. W. Lin J. H. Qian J. P. Zhou J. (2015). Identification of risk factors for intraoperative hemobilia and its correlation with early postoperative complications in patients with hepatolithiasis. Am. J. Surg. 209, 260–267. 10.1016/j.amjsurg.2014.05.032 25190546

